# Keeping the momentum: suggestions for treatment policy updates in the final push to eliminate malaria in India

**DOI:** 10.1186/s12936-023-04558-7

**Published:** 2023-04-18

**Authors:** Neena Valecha

**Affiliations:** Independent Malaria Expert, New Delhi, India

**Keywords:** Malaria case management, WHO malaria guidelines, Indian treatment guidelines, *P. falciparum*, *P. vivax*, Malaria in pregnancy, Severe malaria

## Abstract

Malaria case management with prompt and effective treatment is critical to minimize morbidity and mortality, reduce transmission and to prevent the emergence and spread of anti-malarial drug resistance. India has the highest burden of malaria in South East Asia Region and has made impressive progress in the reduction of the malaria burden in recent years. Since the last revision to the Indian national malaria treatment policy in 2013, guidelines on new treatment strategies have been published for the control/ elimination of malaria by the World Health Organisation (WHO). The most recent update was in March 2023 based on the new evidence available. India’s success is the Region’s success. Therefore, to meet the national as well as regional targets of elimination, the Indian National Programme needs to consider WHO guidelines, deliberate with stakeholders and experts so as to tailor and adapt to the local context, and update National policies to incorporate the relevant ones. Technical aspects of new WHO guidelines which need to be considered for updating India’s treatment policy are discussed.

## Background

India, like other countries in Asia Pacific region aims to eliminate malaria by 2030, in line with the commitment by the Heads of State/Ministers of Health which was most recently re-endorsed in the Ministerial Declaration on Accelerating and Sustaining Malaria Elimination in 2017 [1].

India has made impressive progress towards malaria elimination and has contributed significantly to the decline in malaria burden in the WHO South-East Asia Region in recent years. However, India still contributed 79% of the WHO estimated caseload of 5.4 million cases in the region in 2021. The reported burden in the SEARO region was 0.55 million cases with India contributing 29% of cases [2]. In addition, there remain many technical and operational challenges to reach 2030 elimination goals.

The malaria footprint in India has shrunk considerably in recent years with a 60% reduction in cases from 430,000 in 2018 to 173,975 in 2022. In 2022, four states accounted for 65% of the 173,975 malaria cases in the country (West Bengal (23%), Chhattisgarh (17%), Odisha (14%) and Jharkhand (11%) *Plasmodium falciparum* accounts for 57% of all cases. *Plasmodium vivax* is the predominant species in states closer to malaria elimination, particularly in urban areas as well as in West Bengal [3].

The National Strategic Plan 2017–2022 outlined key strategic approaches based on the malaria endemicity for different states/ districts which are grouped into four categories [4]. Each category should deploy a mix of interventions for vector control, use of long-lasting insecticidal nets, quality diagnosis, case management, and surveillance in line with the local epidemiological situation.

An appropriate treatment policy is the cornerstone for effective case management to cure the patient and interrupt the transmission of malaria. Moreover, the treatment policy must respond to the significant changes in disease dynamics and the microenvironment since 2013 when the malaria treatment policy was last updated. In addition, WHO guidance on case management has evolved in line with new evidence for safer and more effective strategies. The Indian Malaria Programme needs to deliberate on WHO guidance along with national evidence with the technical expert groups and stakeholders to adapt the global guidance to suit the specificity of India and update the guidelines accordingly. The following aspects demand prompt attention.

## Moving to fixed dose combination for treatment for *P. falciparum* malaria for the entire country

Artemisinin-based combination therapy (ACT) is the treatment of choice for *P. falciparum* malaria [5]. In India, combination therapies registered by regulatory authorities for treatment of falciparum malaria are artemether-lumefantrine (AL), artesunate-amodiaquine, artesunate-mefloquine, dihydroartemisinin-piperaquine, artesunate + sulfadoxine-pyrimethamine (AS + SP) and arterolane maleate + piperaquine phosphate. In the National Malaria Programme, AS + SP is the recommended treatment for *P. falciparum* malaria except in Northeast states where artemether-lumefantrine (AL) is recommended since 2013, following reports of treatment failure with AS + SP [6].

AS + SP as well as AL remain effective in the states where they are recommended [7, 8]. However, due to increasing resistance to SP and various other factors listed below, it will be appropriate to replace AS + SP with AL (already used in the Northeast) or another WHO recommended fixed dose ACT in the entire country.

### Sulfadoxine-pyrimethamine (SP) resistance

Mutations in dihydropteroate synthase (DHPS) and dihydrofolate reductase (DHFR) enzymes encoded by malaria parasites may increase due to drug pressure when SP is used for malaria treatment. Recent review by Chaturvedi et al. [9] has also shown that 27% triple mutations (AGEAA, SGEGA) in *P. falciparum* dihydropteroate synthase (*Pfdhps)* are present in Northeast India, followed by single (14%; S436A/F, A437G, K540E, A613S/T) and double (11%; SGEAA, AGKAA, SGKGA) and quadruple mutations (4%; AGEGA) [10–12]. India also has high prevalence of double mutations (57%) in *P. falciparum* dihydrofolate reductase (*Pfdhfr*) followed by triple (16%; C50R-N51I-S108N; N51I-C59R-S108N, N51I-S108N-I164L, C59R-S108N-I164L; quadruple (10%) (N51I-C59R-S108N-I164L), quadruple (10%; (N51I-C59R-S108N-I164L) and single (7%; A16V, C50R, N51I, C59R, S108N, I164L). Double mutations (C50R-N51I, N51I-C59R, N51I-S108N) have been detected in the Northeastern states, Chhattisgarh, Jharkhand, Odisha, West Bengal and Uttar Pradesh [11–17]. Even quintuple (three mutations in dhfr (N51I-C59R-S108N) and two mutations in dhps (A437G-K540E) and sextuple resistance (three mutations in dhfr (IRN) and three mutations in dhps (A437G-K540E-A581G) mutations have been reported from the Northeastern states and a few areas in West Bengal between 2006 and 2013 [18]. Decreased efficacy of partner medicine (SP) due to resistance, can endanger artemisinin, the most valuable tool available for malaria treatment. Therefore, ACT with effective partner medicine should be used. The WHO recommends use of fixed dose combinations for better compliance as well as for preventing monotherapy with either medicine in the combination which could lead to the development of drug resistance [5].

### Lack of efficacy in *P. vivax* and mixed-infections

Uncomplicated *P. vivax* infections can be treated with chloroquine in chloroquine susceptible infections or ACT. However, AS + SP is not recommended by the WHO for the treatment of *P. vivax* malaria as resistance has compromised its efficacy [5]. This is the case in several areas in South-East Asia where *P. vivax* has become resistant to SP more rapidly than *P. falciparum* [5]. This poses a major challenge for the treatment of mixed infections and when patients are incorrectly diagnosed with *P. falciparum* only.

### Contradicted in first trimester of pregnancy

Pregnant women constitute an important risk group for malaria infection, particularly in areas with moderate to high transmission. As AS + SP is not recommended for use in the first trimester of pregnancy, these women cannot be treated for the malaria infection with this ACT [5].

### Impedes the use of SP for IPTp

SP is the WHO-recommended treatment of choice for intermittent preventive treatment of malaria in pregnancy (IPTp). This cannot even be considered in India as long as AS + SP remains the standard treatment [5].

### Contraindicated in infants in first few weeks of life

AS + SP should be avoided in the first weeks of life because it may aggravate neonatal hyperbilirubinaemia [5].

### No child friendly formulations

As swallowing tablets could be a challenging task for children, child-friendly formulations are preferred. AS + SP is not available in a dispersible formulation. In addition, there is no WHO prequalified formulation of AS + SP.

### Contraindicated in patients co-infected with HIV on co-trimoxazole

People who are being treated with co-trimoxazole for HIV AIDS, should not be treated with AS + SP for uncomplicated *P. falciparum* malaria [5].

### Adverse effects

Allergic reactions to sulphonamides are common e.g. Stevens Johnson syndrome [19].

### Increases gametocytes

SP is known to increase gametocytes which can fuel the onward transmission of malaria [20].

## Effective and safer options are available to treat malaria in pregnancy

The current guidelines recommend quinine in first trimester of pregnancy in India [6]. In view of the adverse effects of quinine as well as overall safety, the WHO has recently updated the recommendation in favour of treating malaria in first trimester of pregnancy with an artemisinin-based combination therapy (ACT). In view of the limited data for most artemisinin-based combinations in the first trimester of pregnancy and AS + SP being contraindicated, AL is now the WHO recommended treatment for this patient population [5].

## Lower dose for gametocytocidal treatment for *P. falciparum*

Indian treatment policy currently recommends Primaquine in the dose of 0.75 mg/kg to prevent transmission in *P. falciparum* in line with the 2010 WHO recommendations [6, 21]. In view of the risk of haemolysis in glucose-6-phosphate dehydrogenase (G6PD) deficient patients with the use of primaquine, the WHO now recommends a lower dose of primaquine (0.25 mg/kg) for preventing transmission in *P. falciparum* [5]. The recommendation of lower dose followed the review of studies of transmission-blocking activity based on the infectivity of patients or volunteers to anopheline mosquitoes [22]. This dose can be administered safely without G6PD testing. It will be useful to consider lower dose of primaquine for the India treatment policy.

## Shorter course of treatment for *P. vivax* radical cure

Managing vivax malaria is challenging due to poor adherence to the recommended 14-day course of primaquine as well as the risk of haemolysis in G6PD deficient patients. The Indian guidelines advise caution before administering primaquine in areas known to have high prevalence of G6PD deficiency [6]. In addition, patients need to be advised to stop primaquine immediately if they show signs of haemolysis. Primaquine is provided for the treatment of *P. vivax* at all levels of the health services in India at a dose of 0.25 mg/kg for 14 days. Community health workers (ASHAs) receive incentive for following up patients to help ensure compliance and safety. However, pharmacovigilance at all levels is critical and needs to be made integral part of policy.

As per WHO guidelines, the best practice is to administer primaquine (the only anti-relapse treatment in India) after testing G6PD. WHO has recently recommended a 7-day course of primaquine [5]. With the availability of a quantitative point-of-care G6PD test, G6PD testing could be used before shorter course primaquine radical cure, and studies for the single-dose cure, need to be expedited. This could enhance compliance and also reduce the haemolytic risk with 8-aminoquinolines. However, it will be critical to assess the feasibility of G6PD testing at different levels of the health services to fine tune its incorporation and ensure patient access to safe and effective radical cure.

## Appropriate dose for children with severe malaria

The dose of artesunate in the current guidelines for the management of severe malaria in children under 20 kgs has been modified by the WHO [5] to a higher dose of 3 mg/kg body weight per dose based on pharmacokinetic data. This new recommendation for children needs to be discussed. Parenteral artesunate is the treatment of choice for severe malaria in adults and children. Clinical trials have documented better efficacy and safety of artesunate as compared to parenteral quinine [5, 23]. Artemether, already in the Indian guidelines, is an appropriate alternative if artesunate is not available.

## Interventions for the final phase of elimination

The recent update of WHO guidelines provides recommendations for targeted strategies for specific epidemiological situations including mass drug administration in delimited geographical areas to reduce transmission, mass testing and treatment, and other approaches. These need to be deliberated and tailored to the local context at the subnational level, as required.

## Conclusion

Appropriate and effective case management is crucial in order to achieve the malaria elimination goals. Indian malaria treatment policy was reviewed and last revised in 2013 [6, 24]. WHO’s new evidence-based guidelines will inform Indian policy discussions to select the most appropriate strategies for malaria prevention and case management, adapted to different transmission settings. There are some low hanging fruits, such as adopting a fixed-dose combination treatment for *P. falciparum* malaria for the entire country, updating the dosage for the management of severe malaria, recommending an ACT for management of malaria in the first trimester of pregnancy. Other strategies such as IPTp for pregnant women in high and medium transmission settings and use of mass drug administration (MDA) or mass testing and treatment (MTaT) may require deliberation to tailor the strategies for specific epidemiological settings.

Implementation research should be undertaken to assess the feasibility of G6PD testing patients before shorter course of primaquine or new single dose treatment being developed for radical cure of vivax malaria. Improving the effectiveness of vivax radical cure will help areas that are struggling to control *P. vivax* in challenging settings, such as urban areas, and those that have only a few cases. It is also vital to ensure that surveillance in predominantly vivax areas, tracks progress to the elimination of *P. vivax.*

India is a large country with diverse epidemiology, ecological habitats, and health infrastructure therefore micro-stratification and the use of local data will facilitate the development of tailored strategies and treatment policies to achieve sustainable impact in a given setting. Inputs from states/ districts, stakeholders and experts are needed to analyse and use strategic information and WHO guidance for achieving malaria elimination goal in 2030. Considering that India has highest malaria burden in the SEA, India’s success is critical for bordering countries as well as for the Region.

## Data Availability

Perspective using data from published literature.
